# The Influence of Sonication Processing Conditions on Electrical and Mechanical Properties of Single and Hybrid Epoxy Nanocomposites Filled with Carbon Nanoparticles

**DOI:** 10.3390/polym13234128

**Published:** 2021-11-26

**Authors:** Matheus Mendes de Oliveira, Sven Forsberg, Linnéa Selegård, Danilo Justino Carastan

**Affiliations:** 1 Center for Engineering, Modeling and Applied Social Sciences, Federal University of ABC, Santo André, São Paulo 09210580, Brazil; matheus.mendes@ufabc.edu.br; 2 2D fab, 85350 Sundsvall, Sweden; sven.forsberg@2dfab.se; 3 Saab AB, Business Area Aeronautics, 58188 Linköping, Sweden; linnea.selegard@liu.se

**Keywords:** hybrid nanocomposites, processing, sonication, graphene nanoplatelets, carbon nanotubes, epoxy, electrical conductivity, synergy

## Abstract

Graphene nanoplatelets (GNP) and carbon nanotubes (CNT) are used to enhance electrical and mechanical properties of epoxy-based nanocomposites. Despite the evidence of synergetic effects in the hybrid GNP-CNT-epoxy system, there is still a lack of studies that focus on the influence of different dispersion methods on the final properties of these ternary systems. In the present work, direct and indirect ultrasonication methods were used to prepare single- and hybrid-filled GNP-CNT-epoxy nanocomposites, varying the amplitude and time of sonication in order to investigate their effect on electrical and thermomechanical properties. Impedance spectroscopy was combined with rheology and electron microscopy to show that high-power direct sonication tends to degrade electrical conductivity in GNP-CNT-epoxy nanocomposites due to damage caused in the nanoparticles. CNT-filled samples were mostly benefitted by low-power direct sonication, achieving an electrical conductivity of 1.3 × 10^−3^ S·m^−1^ at 0.25 wt.% loading, while indirect sonication was not able to properly disperse the CNTs and led to a conductivity of 1.6 ± 1.3 × 10^−5^. Conversely, specimens filled with 2.5 wt. % of GNP and processed by indirect sonication displayed an electrical conductivity that is up to 4 orders of magnitude higher than when processed by direct sonication, achieving 5.6 × 10^−7^ S·m^−1^. The introduction of GNP flakes improved the dispersion state and conductivity in hybrid specimens processed by indirect sonication, but at the same time impaired these properties for high-power direct sonication. It is argued that this contradictory effect is caused by a selective localization of shorter CNTs onto GNPs due to strong π-π interactions when direct sonication is used. Dynamic mechanical analysis showed that the addition of nanofillers improved epoxy’s storage modulus by up to 84%, but this property is mostly insensitive to the different processing parameters. Decrease in crosslinking degree and presence of residual solvent confirmed by Fourier-transform infrared spectroscopy, however, diminished the glass transition temperature of the nanocomposites by up to 40% when compared to the neat resin due to plasticization effects.

## 1. Introduction

Epoxy resins are extensively used in high performance applications due to their high chemical resistance, low density and excellent mechanical properties, making them one of the most important classes of thermosetting polymers [1]. For these reasons, epoxy resins became a standard for polymer matrix composites (PMCs), which are widely employed in structural applications [2]. Among PMCs, epoxy is especially important in the fabrication of composites known as fiber-reinforced polymers (FRP), resulting in materials that offer mechanical properties comparable to metals but at a much lower density. The development of such materials revolutionized the aerospace industry by allowing it to switch the fuselage design from metallic to FRP structures, reducing the overall weight of aircraft and saving fuel [1,3]. However, epoxy-based materials have their own set of limitations, including low thermal conductivity that can lead to heat build-up in electronics coated with them [4,5]. Low fracture toughness [2] is another drawback, being one of the main causes of delamination in FRP [6]. Another disadvantage is their inherently high electrical resistance, which is a major source of concern for the aerospace industry [3,7,8]. Airliners are struck by lightning approximately once every year, and the insulating character of modern FRP frames renders the airplane structure vulnerable to serious damage when hit by such electrical discharges, including embrittlement, delamination and vaporization of the resin and metallic components [3,7,8,9]. A conductive fuselage is also required in order to shield the airplane from electromagnetic interference (EMI), which can cause malfunctioning of communication equipment and electronics on-board [3]. So far, the industry uses a metallic wire mesh bonded on the fuselage as an effective solution [3,9], but it is not an efficient solution since it adds considerable weight.

Regarded as “wonder materials” due to their outstanding electrical and mechanical properties, carbon nanotubes (CNT) and graphene, as well as their less costly counterparts such as graphene nanoplatelets (GNP), have enabled researchers to overcome epoxy resins’ limitations by using them as advanced nanofillers [10,11,12]. Recent studies continue to show the beneficial effects of carbon nanofiller addition on epoxy’s properties. Mostovoy et. al. showed that functionalized multi-walled carbon nanotubes significantly increased impact and tensile strength, bending stress and elastic modulus of plasticized epoxy [13]. Hesam et al. was able to improve several mechanical and tribological properties of GNP-epoxy specimens by controlling nanofiller loading and modifying it with silane groups [14]. Lately, hybrid nanocomposite systems (i.e., systems that employ two or more fillers) have been gaining attention and pose a promising alternative to reduce cost and further enhance the range of properties of single-filler nanocomposites [15]. This is due to synergetic effects that may arise when both nanoparticles are mixed together, which are usually justified in terms of better dispersion and bridging of GNP flakes. In the first mechanism, the difference in aspect ratio between GNP and CNT makes it more difficult for particles to re-agglomerate, improving dispersion [16,17]. In the second mechanism, a small amount of CNTs can act as bridges between adjacent graphene flakes and connect them to form a percolating network [16,18,19,20]. In particular, ternary GNP-CNT-epoxy hybrid nanocomposites became the focus of widespread research, but the existence of the much desired synergy in these systems is still far from consensus. In a seminal paper from 2008, Yu et al. have found synergy for thermal conductivity but not for electrical conductivity [4], while a 2013 study by He and coworkers found synergy for both properties [21]. In the following year, Yue et. al. reported synergy for electrical and flexural properties [17]. Then, in 2018, Prolongo and colleagues [22] reported no synergy for glass transition temperature (T_g_), thermal or electrical conductivity, only for storage modulus (E’), while two 2019 papers reported synergy for every property tested, including T_g_ and electrical conductivity [16,23]. Other authors have also discussed these contradictory findings [20,24], which are related to differences in the starting materials and preparation methods. This suggests that more systematic research is needed in order to understand all variables involved, as Navjot points out in a 2019 review paper [25].

Proper dispersion of nanofillers is crucial for achieving the desired properties and could be one of the culprits of these discrepancies, since each study follows a different dispersion procedure. The effect of sonication processing parameters on nanofillers’ dispersion and properties has been studied before. Mellado et al. studied the impact of direct and indirect sonication on the exfoliation and integrity of graphene oxide (GO) and found that high-power direct sonication is more efficient in exfoliating the GO flakes, but it induces defects in the GO sheet structure and therefore sonication bath should be preferred [26]. Sauter and colleagues investigated the influence of amplitude and hydrostatic pressure in the dispersion of silica nanoparticles and found that the de-agglomeration depended only on the total specific energy input [27]. Regarding GNP-epoxy nanocomposites, Silva et. al. compared solvent assisted and non-solvent assisted sonication methods and found that the latter lead to higher electrical conductivity and storage modulus [28]. Despite these previous studies, the impact of sonication parameters on hybrid GNP-CNT-epoxy systems has been ignored so far.

The present work aims to fill this gap by systematically investigating the influence of different sonication parameters and methods in the dispersion, electrical and thermomechanical properties of single and hybrid-filled CNT-GNP-epoxy nanocomposites.

## 2. Materials and Methods

### 2.1. Materials

Aerospace-grade epoxy system Araldite^®^ LY 5052/Aradur^®^ 5052 was purchased from Huntsman (São Paulo, Brazil) and the resin/hardener mass fraction used was 100 to 38, respectively, as recommended by the manufacturer [29]. This epoxy resin is a blend of phenol novolac resin and 1,4 butanediol diglycidyl ether, and the hardener component is a mixture of two amines, IPDA and cycloaliphatic diamine [30]. Graphene nanoplatelets (GNP) were produced and provided by 2D fab (Sundsvall, Sweden). Multiwalled carbon nanotubes (MWCNT) NC7000 were purchased from Nanocyl (Sambreville, Belgium), and the average diameter and length of the tubes are 9.5 nm and 1.5 μm, respectively, with a volume resistivity of 10^−4^ Ω·cm, as stated in the material’s data sheet [31]. For simplicity’s sake, in the present work these will be referred to as “CNT”. Both nanoparticles were used as received.

Weight fractions of 2.5 and 0.25% of GNP and CNT, respectively, were chosen with the purpose of using the minimum amount of nanofiller that would show clear changes in thermomechanical and electrical properties. The concentration of hybrid samples was chosen simply as the combination of the single-filled nanocomposites’ concentrations, i.e., 2.5 wt.% of GNP plus 0.25 wt.% of CNT for a total of 2.75 wt.% of carbon content.

### 2.2. Sample Preparation

For samples dispersed by direct sonication, 40 mL of acetone and the nanoparticles were added in a 50 mL beaker and the nanoparticle suspension was sonicated in the 750 W 20 kHz ultrasonic probe model VCX750 from Sonics (Newtown, CT, USA). The height of the probe was kept constant, and an ice bath was used to prevent overheating. For each of the three compositions (2.5 wt.% GNP, 0.25 wt.% CNT and the 2.75 wt.% hybrid), different combinations of time and amplitude (i.e., power) were used according to Table 1 for a total of 36 samples with different compositions and sonication parameters. After sonication, epoxy resin was added, and the mixture was heated to 70 °C under magnetic stirring to remove the acetone. Then, the samples were put into a vacuum oven at 70 °C overnight in order to further remove the solvent. Control samples were prepared by suspending the nanofillers in acetone and subjecting them to all procedures except the sonication step. For samples processed by indirect sonication, the epoxy resin and nanoparticles were manually mixed for 5 min and then sonicated for 120 min in the 70 W 40 kHz ultrasonic bath model SoniClean 2PS from Sanders (Minas Gerais, Brazil), with manual stirring every 40 min to maintain homogeneity. Acoustic power delivered to the samples was measured through the calorimetric method [32] and found to be ~0.53 W.

**Figure 1:**
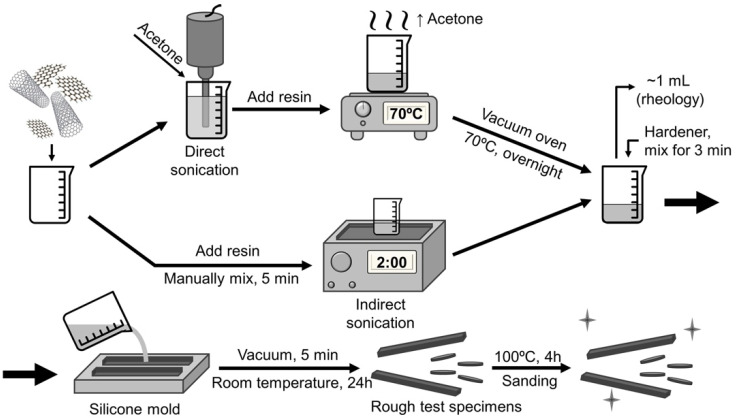
Simplified summary of the sample preparation process.

After both processing routes, samples were taken for rheological characterization before curing, while the remaining material was manually mixed with the hardener for 3 min. The resulting mixture was put under vacuum for 5 min for degassing and then cured for 24 h at room temperature followed by a post-curing step of 4 h at 100 °C. For each condition, two test specimens were prepared for DMA and four for impedance analysis. This procedure is summarized in Figure 1.

### 2.3. Instrumental

Scanning electron microscopy (SEM) was used to image nanoparticles and cured nanocomposites. GNP and CNT powder were dispersed in either dimethylformamide or acetone at approximately 0.1 mg·mL^−1^ and dropped onto a heated silicon wafer substrate. Cured samples were cryofractured and sputter-coated with 15 nm of gold. Images were taken using a compact SEM (JSM-6010LA) or a field-emission SEM (JSM-6701F), both from Jeol (Tokyo, Japan). Small-amplitude oscillatory shear (SAOS) tests were performed at 25 °C in an MCR 502 rheometer from Anton Paar (Graz, Austria). Parameters used include 25 mm diameter parallel plate geometry with 1 mm gap, shear strain amplitude of 1% (within the linear viscoelastic region) and angular frequencies ranging from 0.1 to 100 rad s^−1^. Viscosity curves were also obtained in the same configuration, with shear rate ranging from 0.01 to 1000 s^−1^. Dynamic mechanical analysis (DMA) was performed in a DMA Q800 from TA Instruments (Thermo Fisher Scientific, Waltham, MA, USA) by sweeping temperatures from 35 to 200 °C at a fixed frequency of 1 Hz and 5 µm amplitude in dual cantilever mode. The storage modulus (E’) was evaluated at the glassy state (40 °C) and glass transition temperature (T_g_) obtained as the maximum in tan (δ). Results were averaged using at least two test specimens with dimensions of approximately 12.7 mm × 3.2 mm × 35 mm. Raman spectroscopy was performed on the dispersive Raman T64000 from Horiba Jobin-Yvon (Edison, NJ, USA), with a green laser (532 nm) Verdi G5 from Coherent Inc (Santa Clara, CA, USA), operating at 1 mW. GNP was analyzed as powder (deposited onto a glass slide) and also as individual flakes (suspended in DMF and deposited onto a silicon wafer substrate). Fourier-transform infrared spectroscopy (FTIR) was performed in ATR mode (attenuated total reflectance) with a Spectrum Two (Perkin Elmer, Waltham, MA, USA) instrument, ranging between 500–4000 cm^−1^ at room temperature. Spectra were obtained by a data collection of 32 scans and resolution of 1 cm^−1^. Electrical properties were measured with an SI 1260A gain phase analyzer, coupled with an 1296A dielectric interface, both from Solartron (Leicester, UK). Dielectric spectra were taken from 0.1 Hz to 1 MHz with an applied AC voltage of either 1 or 3 volts, depending on the resistivity of the sample. Specimens were 1 mm thick disc-shaped with about 16 mm of diameter and were coated with 20 nm of gold on both faces in order to minimize contact resistance. AC conductivity was then calculated from the imaginary permittivity at the lowest frequency available (0.1 Hz) using Equation (1):

σ_AC_ = ω ε_0_ ε″ (ω) (1)

in which ω is the angular frequency, ε_0_ is the vacuum permittivity and ε″ (ω) is the imaginary permittivity at the applied angular frequency.

## 3. Results and Discussion

### 3.1. GNP and CNT Characterization

**Figure 2:**
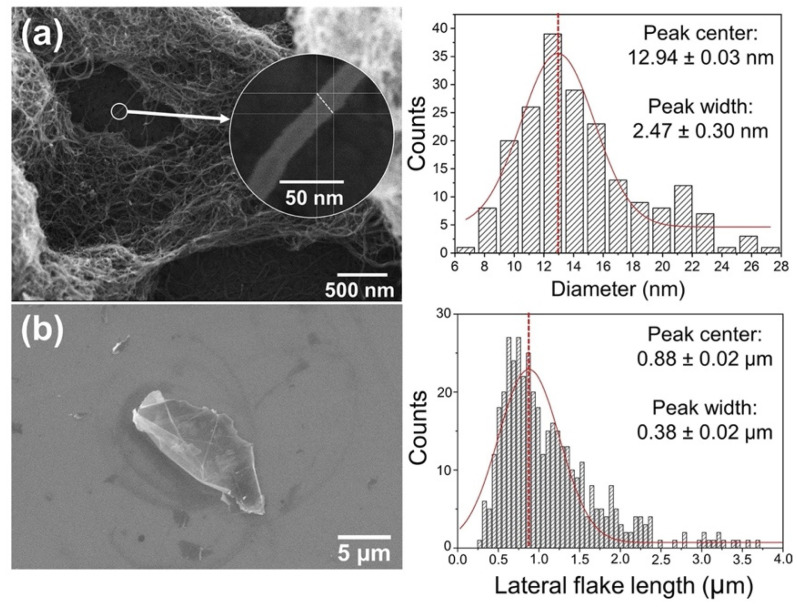
(**a**) FE-SEM image of a CNT bundle and their respective diameter size distribution; (**b**) SEM image of GNPs and their respective lateral size distribution.

The diameters of about 200 individual nanotubes were measured using field-emission scanning electron microcopy (FE-SEM). Figure 2a shows that the gaussian distribution peaked at 12.9 nm, which is close to the mean diameter informed by the manufacturer (9.5 nm) [31]. Multiple SEM images of the GNPs were taken in order to measure about 400 individual flakes and build the lateral size distribution shown in Figure 2b, following the recommended method described by the National Physical Laboratory (London, UK) [33]. Peaking at 0.876 µm, the lateral dimensions are within the expected range for GNPs (100 nm to 100 µm [34]). The distribution width is also adequate for GNPs, since they are known to contain graphene-related materials with a wide range of sizes (from few-layer graphene to nanostructured graphite [35,36]).

**Figure 3:**
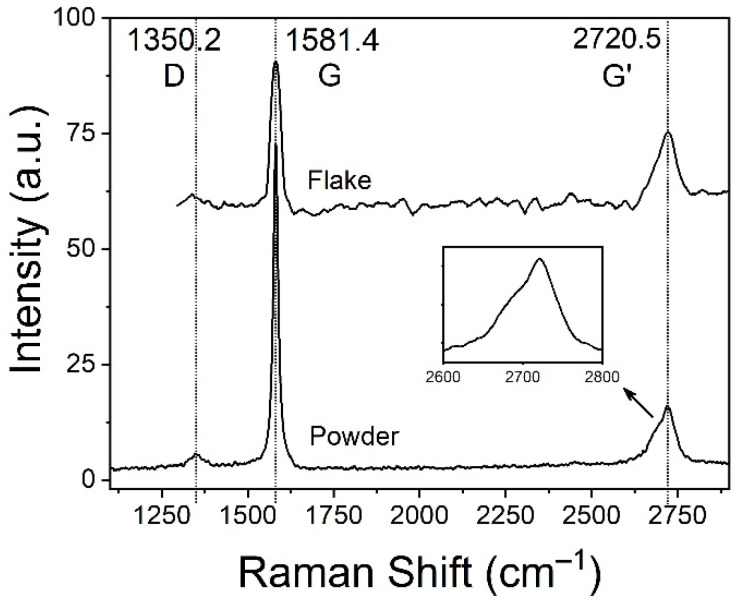
Raman scattering spectra for the as-received GNP and its D, G and G’ bands. Top spectrum was taken from a representative individual GNP flake, and bottom spectrum from GNP powder.

Raman spectroscopy shown in Figure 3 provides more insight about the GNP’s inner structure. The most pronounced band around 1582 cm^−1^ (commonly referred to as “G band”) is associated with in-plane vibrations from sp^2^-hybridized carbon atoms, while the D band around 1350 cm^−1^ is associated with sp^3^-hybridized carbon [37]. The peak intensity ratio I_D_/I_G_ is often used to evaluate the level of disorder in the lattice, which comes from vacancies, kinks, heptagon-pentagon pairs, heteroatoms and other impurities [38,39,40]. Figure 3 shows that I_D_/I_G_ ratio of a representative individual flake is small (<0.2), which suggests a highly ordered carbon crystalline structure.

The shape of G’ band around 2720 cm^−1^ is especially sensitive to the number of graphene layers. For monolayer graphene the G’ line is a symmetrical band that can be fitted with a single Lorentzian peak. However, this band splits into four components in bi-layer graphene, which causes a broadening of the resulting peak along with a slight upshift. With an increasing number of layers both effects escalate, and above 5 layers the G’ band becomes almost indistinguishable from that of bulk graphite [41]. Shape and position of the G’ band obtained clearly confirms that the GNP flakes are more than 5 layers thick (insert in Figure 3), as expected for this kind of nanomaterial.

### 3.2. Rheology and Electrical Conductivity

**Figure 4:**
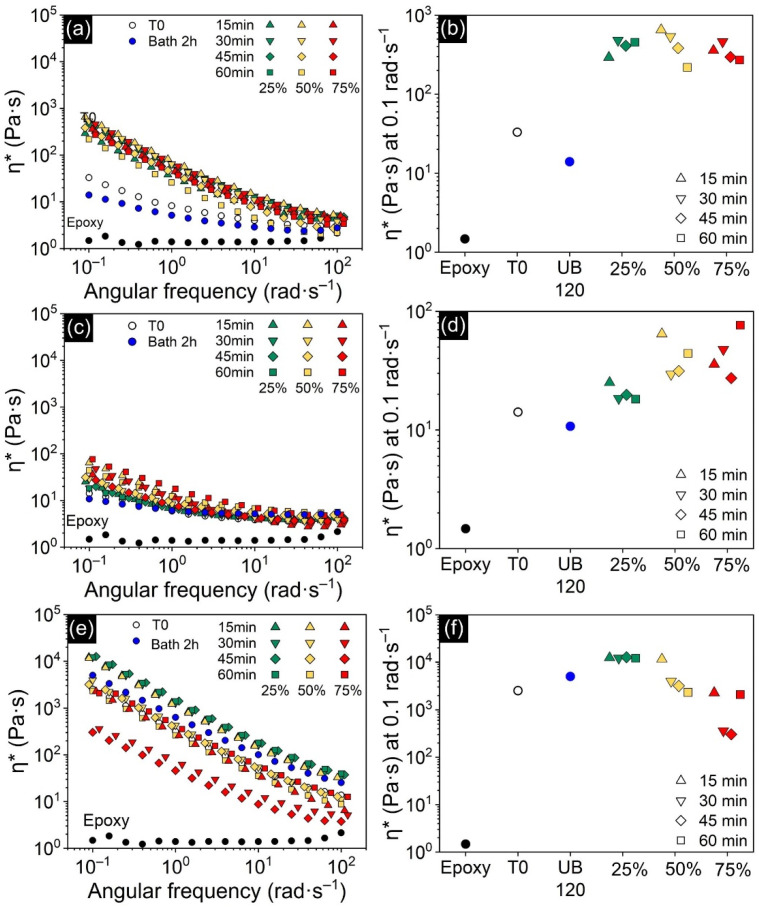
(**a**,**c**,**e**) Complex viscosity (η*) of CNT-epoxy, GNP-epoxy and hybrid-epoxy suspensions, respectively; (**b**,**d**,**f**) show η* taken at 0.1 rad·s^−1^ as a function of processing method. T0 denotes the unprocessed samples and UB 120 the samples processed in the ultrasonic bath.

Electrical conductivity in nanocomposites reinforced with conductive particles is closely related to the formation of a percolating network, which in turn can be assessed by rheological measurements. For this reason, rheological and electrical results are discussed simultaneously. Uncured samples were subjected to small amplitude oscillatory shear tests, in which an increase in complex viscosity (η*) is a sign of dispersion enhancement while a decrease can be interpreted as the predominance of agglomerates [42]. Figure 4a,b show that the addition of 0.25 wt.% of CNTs caused a dramatic increase in viscosity when compared to neat epoxy resin, even at such a low concentration. In addition to the increase in η*, there was also a shift from an essentially Newtonian behavior shown by neat epoxy resin to a shear-thinning behavior of the filled samples, typical of suspensions containing nanofillers [43]. The effect of processing is also clear. The viscosity of the sample processed by an ultrasonic bath dropped when compared to the non-sonicated sample, suggesting a less efficient dispersion, while direct sonication had a positive effect on distribution. However, each amplitude had a different response: viscosity increased and stabilized with time when lower power (i.e., 25% amplitude) was used, but the more intense sonication at 50 and 75% amplitudes caused the viscosity to decrease over time. The decrease can be attributed to shortening of the CNTs, commonly caused by fluid friction at their surface when stronger cavitation bubbles implode nearby [44,45]. The decrease in length impairs the CNTs’ ability to form percolated networks [46], and thus better-connected structures were achieved when using direct sonication at low power, or shorter times with moderate power.

**Figure 5:**
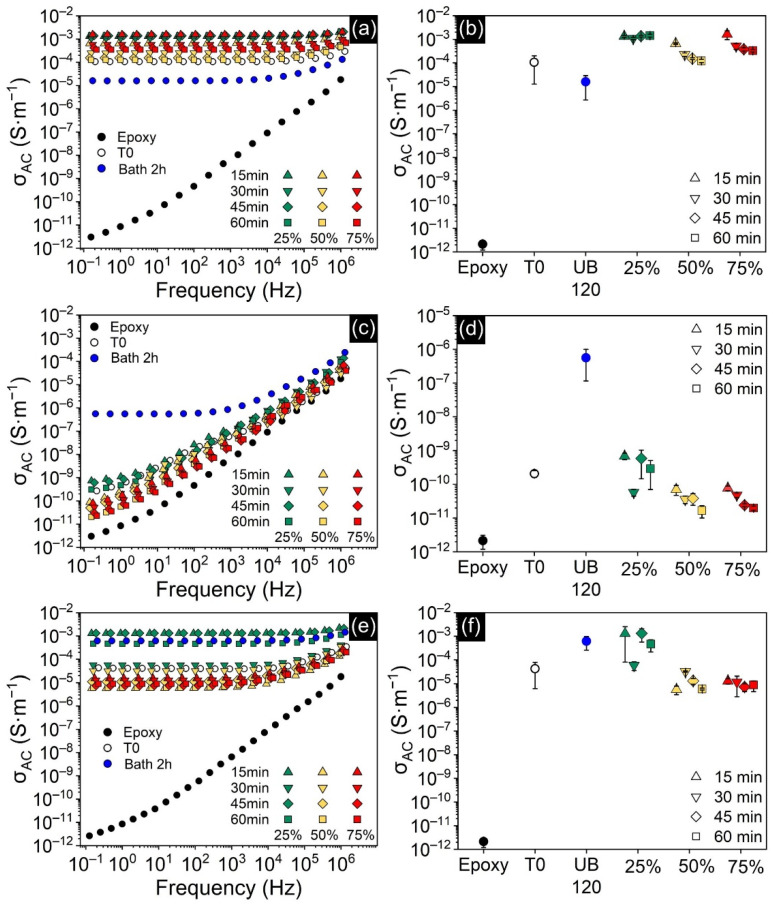
(**a**,**c**,**e**) σ_AC_ spectra of CNT-filled, GNP-filled and hybrid-filled nanocomposites, respectively; (**b**,**d**,**f**) σ_AC_ taken at the lowest frequency (0.1 Hz) as a function of processing method. T0 denotes the unprocessed samples and UB 120 the samples processed in the ultrasonic bath.

Figure 5a displays σ_AC_ spectra for CNT-filled nanocomposites and neat epoxy. The AC conductivity measured for neat epoxy confirmed that it is in fact an insulating material. Additionally, its σ_AC_ spectrum shows a strong dependence on frequency over the entire range scanned, another piece of evidence of its insulating character and a conduction dominated by non-ohmic mechanisms [28]. Addition of CNTs at only 0.25 wt.% greatly increased σ_AC_, with samples displaying values up to 10 orders of magnitude higher than neat epoxy. Their σ_AC_ spectra is also drastically different, with σ_AC_ almost completely independent from frequency, indicating that they are above the percolation threshold, i.e., a conductive percolating network is achieved and ohmic conduction is predominant [28]. Comparing Figure 4b and Figure 5b reveals that dispersion state assessed by rheology correlates remarkably well with the electrical conductivity of cured samples, confirming that better-connected networks translated into superior electrical performance [47]. Samples processed by direct sonication showed better results in this regard, especially at 25% amplitude, managing to maintain high conductivities for all times tested. However, these percolated networks are delicate and tend to degrade fast when sonicated at higher powers, returning to values close to that of the non-sonicated sample.

**Figure 6:**
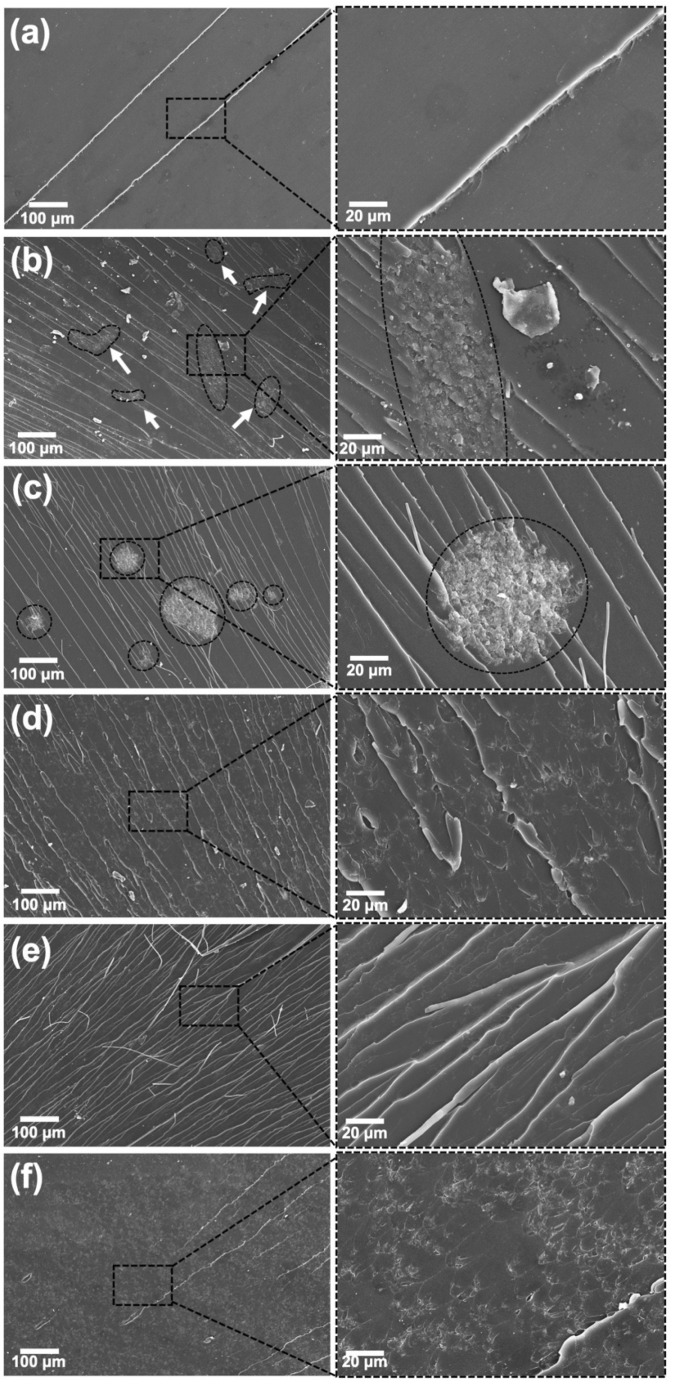
(**a**) SEM images of the cryofractured neat epoxy at different magnifications. Smooth fractured surfaces are typical of brittle-like failures; (**b**) CNT-filled nanocomposites at 0.25 wt.%, prepared without sonication; (**c**) with indirect sonication for 120 min; and with direct sonication for 60 min at 25% amplitude (**d**), 50% amplitude (**e**) and 75% amplitude (**f**). CNT bundles are highlighted.

Rheological and electrical results show that indirect sonication did not assist the formation of effective CNTs networks and produced inferior results than the non-sonicated sample. Figure 6 shows SEM images from cryofractured CNT-filled samples and confirms that the ultrasonic bath was unable to dissolve the CNT aggregates, which stayed entangled in large, spherical bundles (Figure 6c). This unfavorable morphology prevented CNTs from percolating. On the other hand, the non-sonicated sample (Figure 6b) produced small, and more elongated CNT bundles that are more effective at forming a long-range percolated network. Thus, surprisingly, the simple fact of suspending CNTs in acetone before mixing with epoxy resin was more beneficial to dispersion than directly mixing them with epoxy and then sonicating for 120 min in the ultrasonic bath. Figure 6d–f show the effect of direct sonication for each amplitude after 60 min of processing, in which CNT bundles seen in previous samples vanished and the filler is more equally distributed throughout the matrix.

Figure 4c shows that GNPs did not raise viscosity as much as CNTs did, despite the 10 times increase in loading. This is due to the difference in geometry: rod-like nanoparticles can form networks more readily than sheet-like nanoparticles [48,49]. GNP-epoxy suspensions did not seem to benefit from indirect sonication, since η* was almost the same as that of the unprocessed sample, indicating a similar degree of dispersion. In contrast to CNT samples (Figure 4b), the effect of direct sonication on the GNP’s network was less clear. This might be due to the fact that layered materials can develop competing effects with dispersion at high energies of sonication. At first, loosely attached agglomerates are broken down, leading to a better dispersion (and higher η*), but further sonication leads to lateral breaking and/or exfoliation of the GNP sheets [26]. These have opposite effects on the flakes’ aspect ratio, which impact the network formation measured by η*. At 25% amplitude, a 15 min sonication produced the highest η*, while longer times only diminished it. This indicates that the power is not enough to exfoliate the GNP sheets and lateral breaking is predominant after 30 min and beyond. At 50 and 75% amplitude, however, the power seems to be high enough to exfoliate the sheets and raise η* after longer times. Although this effect is always competing with lateral breaking, the general trend is that η* increased with time and amplitude.

Unlike CNT-epoxy samples, better dispersion state in GNP-epoxy suspensions did not translate into higher electrical conductivity. While Figure 5d confirms that GNP-filled nanocomposites’ conductivity was highly dependent on the processing methods, the trend is different from what was observed for η*. Gentler, indirect sonication achieved the highest conductivity of the series: over 5 orders of magnitude higher than neat epoxy and 3 orders of magnitude higher than the non-sonicated sample, reaching 5.6 × 10^−7^ S·m^−1^. On the other hand, σ_AC_ dropped by up to four orders of magnitude for samples processed by direct sonication at 25% amplitude. The effect was even stronger for 50 and 75%, in which longer times invariably deteriorated this property. The σ_AC_ spectra in Figure 5c also revealed that, while all other samples displayed a strong dependence on frequency (typical of insulating materials), the specimen sonicated in the ultrasonic bath exhibited a low-frequency independent behavior characteristic of ohmic conduction. As discussed extensively in the literature, direct sonication can induce the formation of defects in graphene sheets [26,50,51,52] and these defects are known to degrade their sp^2^ structure into sp^2^-sp^3^ with less π-π stacking stability, impairing electrical conductivity [53,54]. This explains why this property deteriorated with higher sonication energies, while processing with ultrasonic bath managed to preserve it. Based on these results, σ_AC_ proved to be more sensitive to the GNP’s sheet integrity than to its dispersion state, and, therefore, avoiding damage to the flakes is a more effective strategy than using stronger sonication if σ_AC_ is the key property.

**Figure 7:**
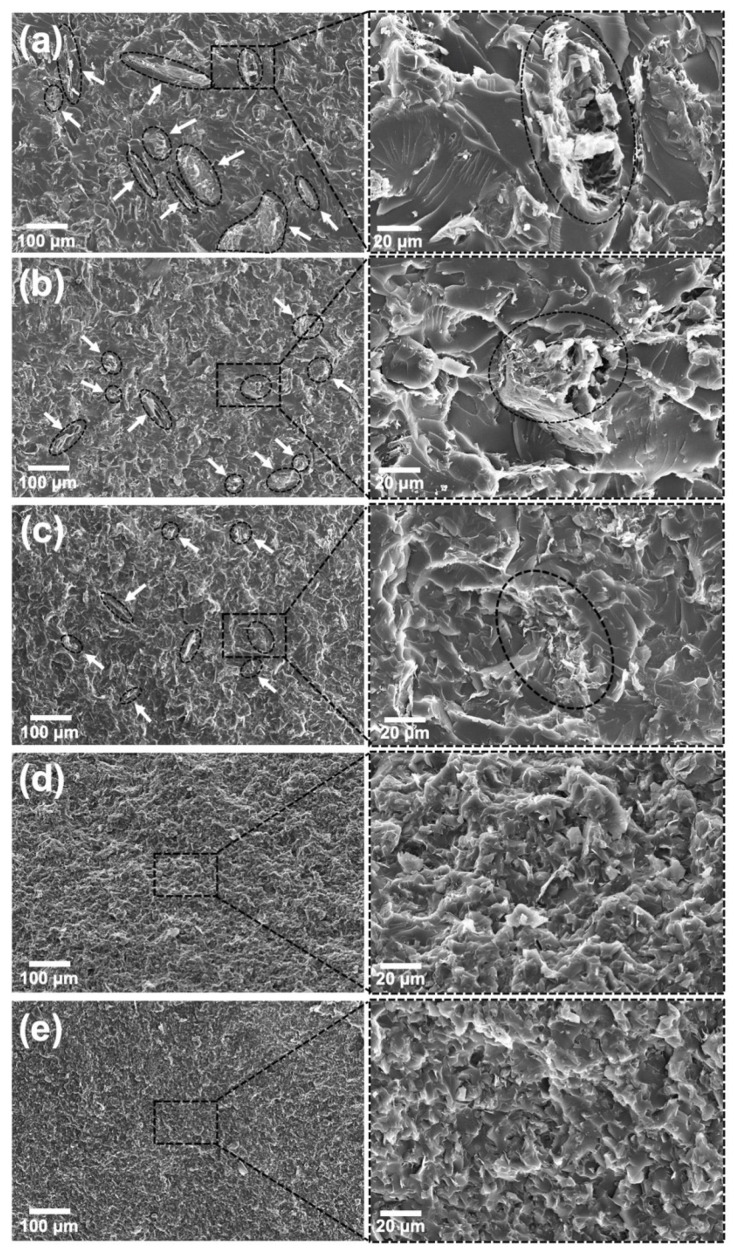
SEM images of GNP-filled nanocomposites at 2.5 wt.% loading, prepared without sonication (**a**); with indirect sonication for 120 min (**b**); and with direct sonication for 60 min at 25% amplitude (**c**), 50% amplitude (**d**) and 75% amplitude (**e**). GNP aggregates are highlighted.

**Figure 8:**
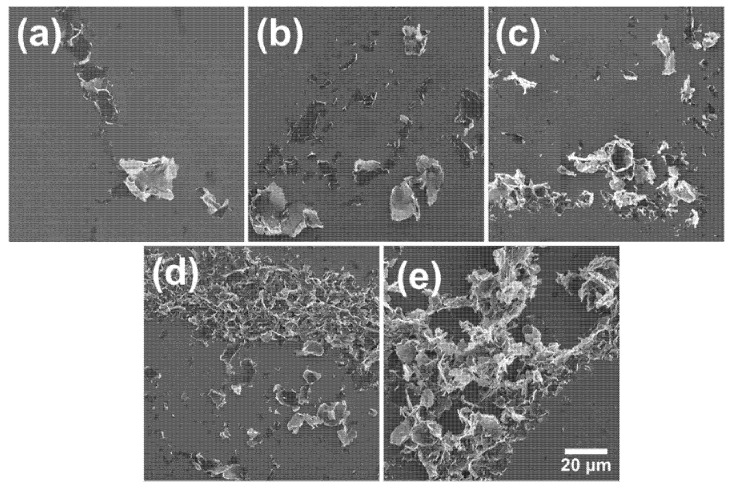
(**a**) Non-sonicated GNP; GNP processed by ultrasonic probe at 75% amplitude for 15, 30, 45 and 60 min ((**b**–**e**) respectively).

Figure 7 shows SEM images of GNP-filled nanocomposites, in which the presence of the GNP flakes at 2.5 wt.% completely altered the smooth fracture surfaces seen in neat epoxy (Figure 6a). Micrographs confirm that higher power produced better dispersed samples, in agreement with η* measurements. The unprocessed nanocomposite displayed many agglomerates (arrows in Figure 7a), while the sample processed by indirect sonication had smaller agglomerates but still maintained the flakes’ lateral dimensions unharmed. Agglomerates seen in the sample prepared by direct sonication at 25% are even smaller, but this time flakes are also significantly shorter, showing that the negative effect is already present. Samples sonicated at 50% amplitude continued the trend and did not display large aggregates, with GNPs finely distributed throughout the matrix, and at 75% the effect is even more pronounced. However, in addition to the reduction in size, GNP flakes are also damaged, with apparent sharper edges and kinks. Figure 8 shows SEM images taken from GNPs processed by ultrasonic probe for 15, 30, 45 and 60 min at the highest power tested (75% amplitude). Although unprocessed flakes look similar to those processed for 15 min, the damage from 30 min and above is clear, with flakes becoming smaller, more wrinkled and with sharper, cracked edges.

The first feature that stands out in the case of hybrid suspensions (Figure 4e,f) is that η* reached values that are orders of magnitude higher than single-filled composites. Although this could be explained by the fact that total nanofiller concentration is higher, most likely this is evidence that a better dispersion was achieved: the difference in aspect ratio between GNP and CNT makes it more difficult for particles to re-agglomerate, improving their distribution in the matrix [16,17]. The effect of sonication on dispersion state of the hybrid samples is similar to that observed for CNT-filled suspensions, but the hybrid nanofiller network formed is even more sensitive to higher energies. While indirect sonication and direct sonication at 25% amplitude resulted in a better-connected network, at 50% it rapidly degraded when the sample was processed for more than 15 min. At 75% amplitude, η* dropped to lower values than that shown by the non-sonicated suspension, although a jump in η* is seen after sonicating for 60 min. The fact that long-duration sonication induces sheet exfoliation [26] can explain this jump in η* after 60 min.

**Figure 9:**
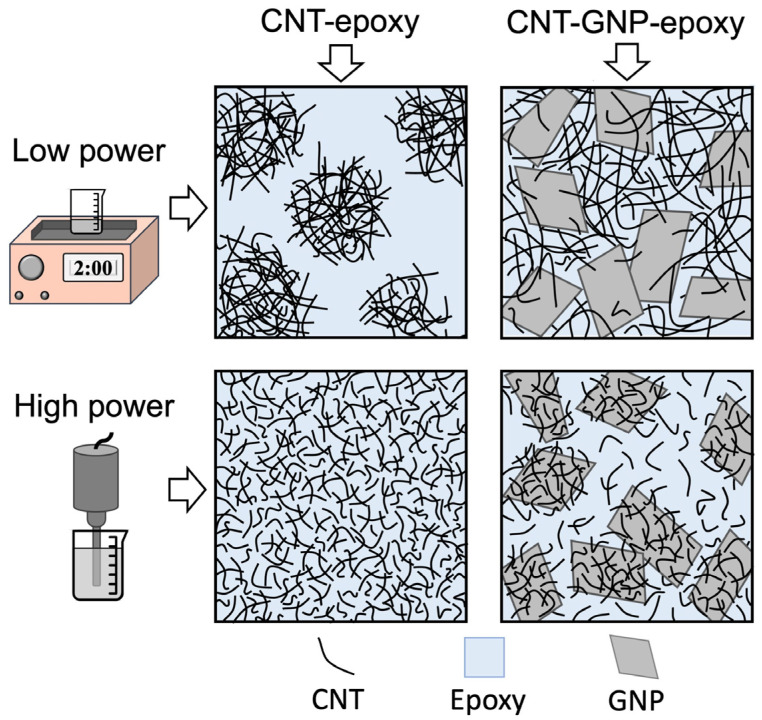
Schematic of the opposing effects of adding GNPs on the CNT network formation. Low-power sonication is not able to break the CNT bundles alone, but the presence of GNP flakes helps reduce bundle size and better distribute them throughout the matrix. With high-power sonication the CNTs become shorter, disentangled and better dispersed, but then tend to be collected onto the surface of GNP flakes due to π-π interactions, impairing their ability to form a percolating network.

Hybrid nanocomposites also achieved σ_AC_ orders of magnitude higher than neat epoxy, and σ_AC_ spectra in Figure 5e display the frequency-independent behavior of a percolated network. However, the highest σ_AC_ recorded for this system (1.3 × 10^−3^ S·m^−1^) was virtually the same as that achieved by CNT-filled samples (1.6 × 10^−3^ S·m^−1^). This reveals that the presence of GNPs in the hybrid did not help increase the maximum σ_AC_. The reason behind it is that CNTs at 0.25 wt.% loading had already established a percolating network on their own, and thus the introduction of GNPs was ineffectual. Prolongo et. al. reached a similar conclusion in a related study [22]. However, in order to properly assess synergism in this system it is necessary to investigate multiple concentrations and loading combinations, which is not the focus of the present work. Regarding the effect of processing, Figure 5f shows that σ_AC_ results are in perfect agreement with the dispersion state evaluated by η*, and thus σ_AC_ is closely correlated to the formation of an efficient percolating network. Hybrid specimens exhibited the highest conductivity (1.3 × 10^−3^ S·m^−1^) when processed with low-power direct sonication at 25% amplitude. However, processing at 50 and 75% amplitude degraded the property, causing it to drop by almost three orders of magnitude. This adverse effect of high-energy sonication is more pronounced in the hybrid specimens than in samples filled with only CNT. At the same time, the hybrid sample processed by the ultrasonic bath improved σ_AC_ by 1 order of magnitude when compared to its CNT-filled counterpart. An important question thus arises: how could the presence of GNPs have a positive effect on σ_AC_ and η* for indirect sonication, while at the same time lead to rapid decreases in both properties for high-power direct sonication? The proposed mechanism behind it is that the presence of GNPs helped disperse and space the large CNT agglomerates that could not be broken by indirect sonication, improving their distribution and contributing to the formation of a percolated network. However, with high-energy sonication, disentangled CNTs become shorter, gain mobility and, due to the strong π-π interaction with graphene sheets, become adhered onto the GNP surface [55]. This diminishes the effective CNT concentration in the epoxy matrix (Figure 9) and impairs percolation.

**Figure 10:**
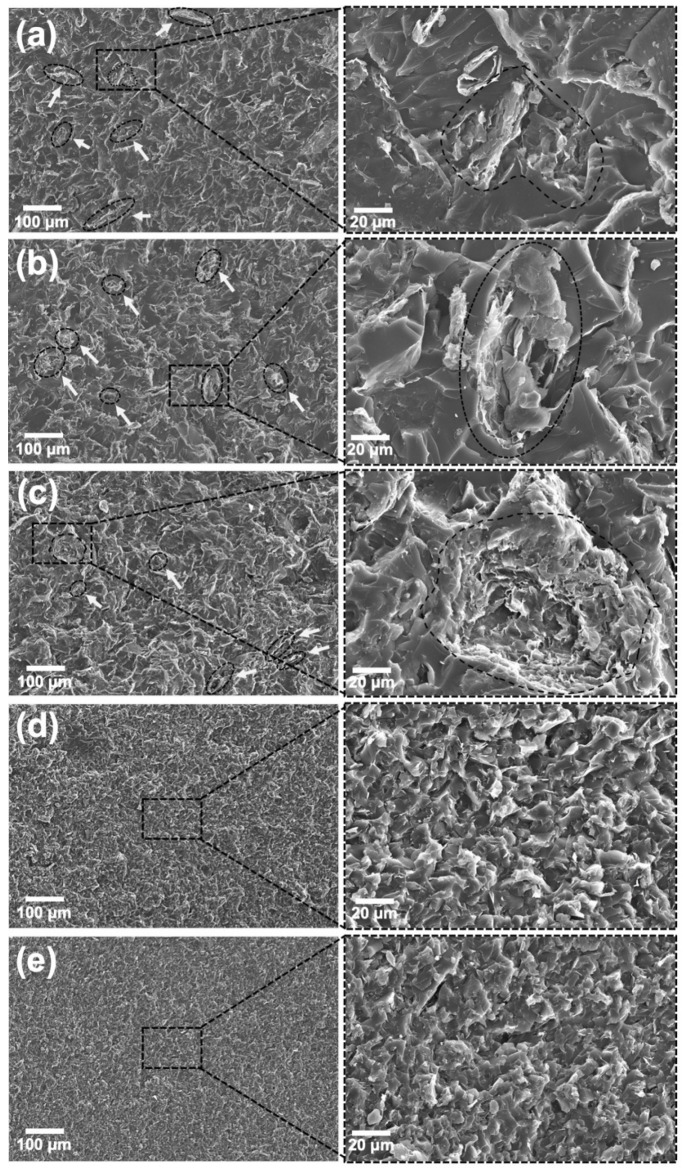
(**a**) SEM images of hybrid nanocomposites loaded with 2.5 wt. % of GNP and 0.25 wt. % of CNT, prepared without sonication; (**b**) with indirect sonication for 120 min; (**c**) with direct sonication for 60 min at 25% amplitude; (**d**) 50% amplitude; (**e**) and 75% amplitude. GNP agglomerates are highlighted.

SEM micrographs in Figure 10 show that the morphology of fractured hybrid samples is similar to that of the GNP-filled nanocomposites. Indirect sonication for 120 min managed to decrease the number of GNP agglomerates (highlighted by arrows) when compared to the non-sonicated sample, and at the same time avoid damage to GNP flakes. The absence of large CNT aggregates in these samples confirms that the steric hindrance caused by the presence of GNPs helped disperse CNT bundles [16,17] and supports the proposed mechanism in Figure 9.

### 3.3. Dynamic Mechanical Analysis (DMA)

**Figure 11:**
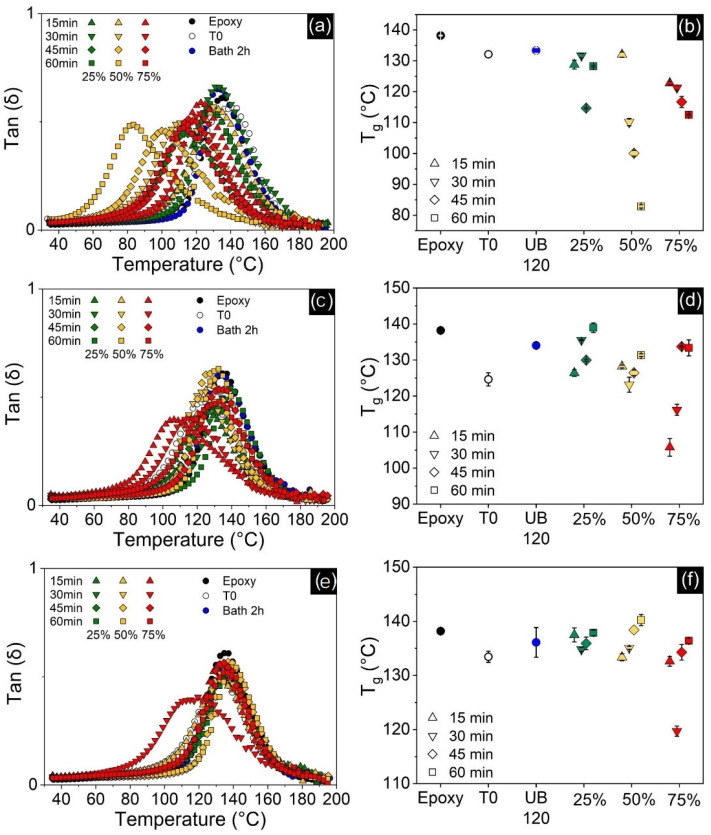
Tan (δ) curves (**left**) and T_g_ results (**right**) for all nanocomposites compared to neat epoxy and as functions of processing methods. CNT-filled at the top (**a**,**b**); GNP-filled in the middle (**c**,**d**); and hybrid-filled nanocomposites at the bottom (**e**,**f**). T0 denotes the unprocessed samples and UB 120 the samples processed in the ultrasonic bath.

**Figure 12:**
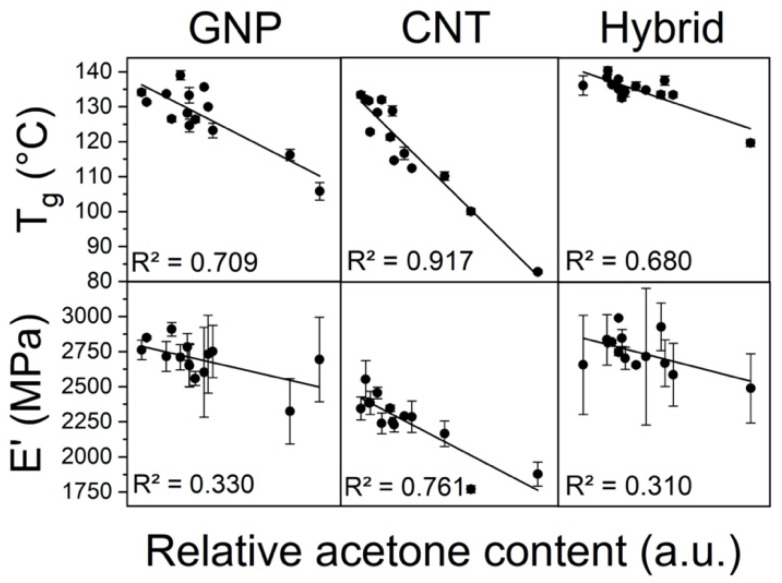
Dependence of T_g_ and E’ on residual acetone content. Straight lines represent linear fitting of the data.

While most studies have reported increases in T_g_ for single-filled CNT [22,23] and GNP [28,56,57] nanocomposites, results in Figure 11b,d show that the addition of these nanofillers did not improve T_g_ when compared to neat epoxy. CNT-filled samples recorded up to 55 °C drops in T_g_, while GNP-filled specimens showed up to 32 °C drops. Hybrid specimens have also shown T_g_ decreases for most processing methods (Figure 11f), although samples processed by direct sonication for 45 and 60 min at 50% amplitude managed to slightly increase it by up to 2 °C. Usually, the higher the filler content and the better the dispersion, the more effective is the hindering of polymer chains and T_g_ is enhanced. However, for thermosetting polymers such as epoxy, the addition of carbon fillers might lead to lower crosslinking density due to a decrease in fluidity, which prevents diffusion of reactive groups during the curing process and causes T_g_ to decrease [58]. Another important factor is residual acetone, which causes a plasticization effect that can strongly decrease T_g_ [59]. Therefore, the final impact on T_g_ is a combination of all these individual contributions. FTIR spectroscopy was performed in order to assess the presence of residual solvent. Figure S1 shows that a measurable amount of acetone is still present in all samples processed by the solvent-assisted sonication method, despite the efforts in eliminating acetone from the system. Plotting the integrated area of characteristic ketone peak (1710 cm^−1^) versus T_g_, a clear dependence arises for both GNP- and especially CNT-filled composites, confirming that residual acetone is in fact the main reason why T_g_ decreased (Figure 12). Since the samples processed by the ultrasonic bath have no acetone, the slight decrease in T_g_ should be due to a reduction in crosslinking density, as discussed previously. Although the acetone-T_g_ correlation also exists for hybrid specimens, they were less affected by it and managed to perform close to neat epoxy. This might be explained by the higher total nanofiller content present in these hybrid systems, which further reduced the matrix free volume and countered the detrimental effect of residual solvent [60].

**Figure 13:**
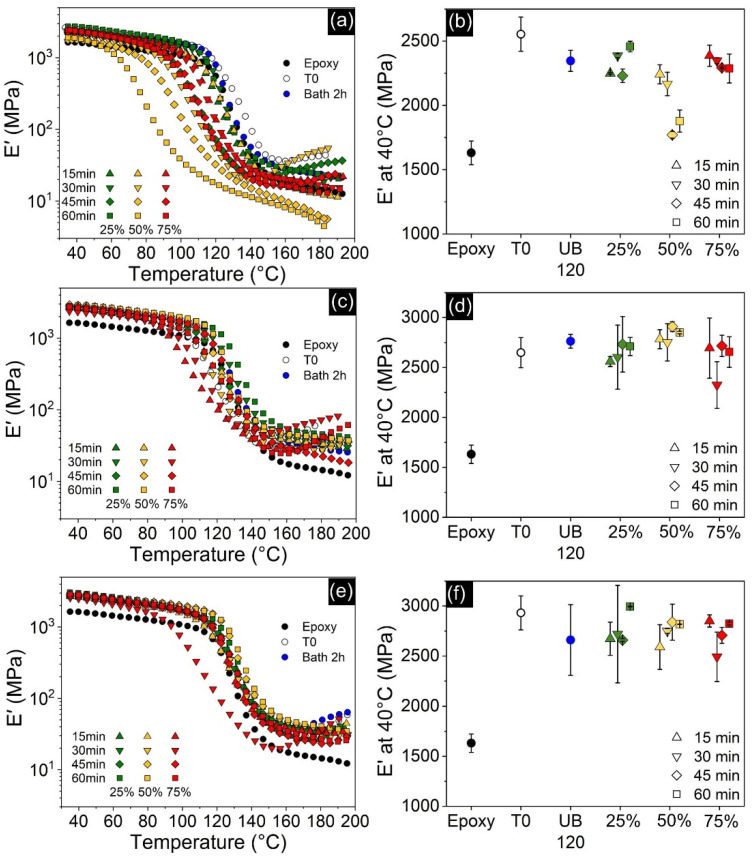
E’ curves (**left**) and E’ at the glassy state (**right**) for all nanocomposites compared to neat epoxy and as functions of processing methods. CNT-filled at the top (**a**,**b**); GNP-filled in the middle (**c**,**d**); and hybrid-filled nanocomposites at the bottom (**e**,**f**). T0 denotes the unprocessed samples and UB 120 the samples processed in the ultrasonic bath.

Although storage modulus (E’) is not identical to Young’s modulus (E), they are conceptually similar and E’ is frequently used to evaluate stiffness of viscoelastic materials [28,61]. Overall, the addition of nanofillers greatly improved E’ for all compositions. This is usually attributed to the high stiffness of the nanofillers, as well as good interfacial interactions that promote stress transfer between matrix and nanoparticles [62,63,64]. Regarding GNP-filled nanocomposites, Figure 13d shows that the different processing parameters had little effect on E’ and, therefore, it is not as dependent on dispersion state. This is an unexpected result, considering the differences in morphology (Figure 7) and η* (Figure 4) achieved by each processing route, and the fact that literature argues that the structure of networks and rheological percolation are related to better mechanical properties [65]. Figure 12 shows that, although the residual acetone content also affected this property, the dependence is rather weak. Notwithstanding, there are still some remarks to report: the specimen processed by indirect sonication achieved an E’ ~69% higher than neat epoxy, which was slightly above the non-sonicated sample and higher than most nanocomposites sonicated by the ultrasonic probe with 25 and 75% amplitudes. However, specimens directly sonicated at 50% amplitude exhibit the highest values of E’ for all times tested, and the sample sonicated for 45 min at 50% amplitude achieved a remarkable 78% improvement over neat epoxy. Therefore, this condition seems to be the best compromise between lateral breaking and further exfoliation of the GNP flakes. Addition of CNT also led to a significant increase in E’, as shown in Figure 13b, reaching up to 57% improvements. Different from GNP-filled samples, however, the results varied considerably for each condition, and Figure 12 shows that this is correlated with residual acetone content. Similar to its effect on T_g_, plasticization decreased the stiffness of the material in proportion to the amount of residual solvent present. Hybrid GNP-CNT nanocomposites have shown even greater increases in E’, with the sample processed at 25% amplitude for 60 min reaching 3000 MPa, an ~84% enhancement over neat epoxy and the highest achieved by all cured specimens. Despite this relevant improvement, it is still below the expected increase based on the separate contribution of each nanofiller given by the rule of mixtures, and thus this specific configuration showed no synergy for E’. Figure 13f shows that, similar to the GNP-filled samples, E’ proved to be little dependent on the different processing methods and residual solvent content. This similarity might be explained by the fact that the morphology of hybrid-filled nanocomposites (Figure 10) closely resembles that of the GNP-filled samples (Figure 7), with little influence of the presence of CNTs.

## 4. Conclusions

- In general, direct sonication led to better dispersion of GNP and CNT nanoparticles than indirect sonication. The better dispersed CNT-filled samples invariably achieved higher electrical conductivities. However, this high-energy method induced defects in the GNP sheets that impaired their electrical conductivity;
- For the GNP-epoxy system, the use of the ultrasonic bath led samples to achieve σ_AC_ four orders of magnitude higher than specimens that were direct-sonicated, despite their inferior dispersion state. Therefore, the sheets’ integrity should be prioritized over the dispersion quality in order to achieve higher electrical conductivity;
- In the hybrid system, the addition of GNP helped improve CNT’s dispersion state when processed by indirect sonication due to the steric hindrance effects. This raised σ_AC_ by almost two orders of magnitude when compared to single CNT-filled samples that were also processed by indirect sonication;
- At the same time, the presence of GNPs in the hybrid caused electrical conductivity to decrease when compared to single CNT-filled samples if high-energy direct sonication is used instead. The proposed mechanism to explain this decrease in σ_AC_ for direct-sonicated hybrid specimens involves selective localization of shortened CNTs onto the GNP flakes due to strong π-π interactions, impairing percolation;
- DMA results showed that, while the introduction of nanofillers significantly improved E’ for all compositions, these enhancements were little impacted by the different sonication methods and parameters. The only relevant fluctuations in E’ happened for CNT-filled specimens processed by direct sonication that were caused by the presence of residual acetone, which led to a plasticization effect;
- T_g_ results were strongly impacted by the plasticization effect caused by residual solvent content, although reduction in crosslinking density also contributed to a lesser degree. Therefore, the use of solvent-assisted methods must be carefully considered when designing the processing procedure, and solvent-free alternatives should be prioritized whenever possible.

## Figures and Tables

**Table 1:** Sonication parameters used for preparing GNP-, CNT- and hybrid-filled nanocomposites.

Sonication Method	Amplitude (%)	Time (min)
Indirect sonication	-	120
Direct sonication	25	15
	25	30
	25	45
	25	60
	50	15
	50	30
	50	45
	50	60
	75	15
	75	30
	75	45
	75	60

## Data Availability

The data presented in this study are available in this article.

## Supplementary Materials

The following are available online at https://www.mdpi.com/article/10.3390/polym13234128/s1, Figure S1: FTIR spectra for all samples, Table S1: Total acoustic energy delivered during sonication and results for each property tested.

## Abbreviations

ATR	Attenuated total reflectance
CNT	Carbon nanotube
DMA	Dynamic mechanical analysis
E	Young’s modulus
E’	Storage modulus
FE-SEM	Field-emission scanning electron microscopy
FRP	Fiber-reinforced polymer
FTIR	Fourier-transform infrared spectroscopy
GNP	Graphene nanoplatelet
IPDA	Isophorone diamine
PMC	Polymer matrix composite
SAOS	Small-amplitude oscillatory shear
SEM	Scanning electron microscopy
T_g_	Glass transition temperature
η*	Complex viscosity
σ_AC_	AC electrical conductivity
