# Supplementary material for: The Influence of Sonication Processing Conditions on Electrical and Mechanical Properties of Single and Hybrid Epoxy Nanocomposites Filled with Carbon Nanoparticles
Source: Polymers (Basel). 2021 Nov 26;13(23):4128. doi: 10.3390/polym13234128 (PMC8659569; doi:10.3390/polym13234128)
Supplement: Supplementary file 1 [file polymers-13-04128-s001.zip › polymers-1461666-supplementary.pdf]

polymers

MDPI

**Supplementary Material**

**Figure S1.** FTIR spectra for all samples. Dashed lines at 1712 cm$^{-1}$ highlight the characteristic ketone band.

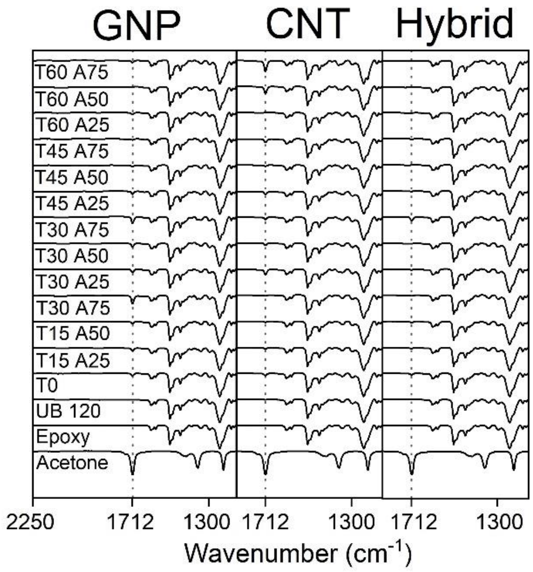


**Table S1.** Total acoustic energy delivered during sonication and results for each property tested. Processing parameters are denoted in samples' names: minutes of processing time after letter "T", and amplitude (%) after letter "A". "UB" stands for ultrasonic bath.

| Sample | Acoustic energy (J) | $\sigma_{AC}$ (S·m$^{-1}$) | E' at 40 °C (MPa) | $T_g$ (°C) |
|---|---|---|---|---|
| Neat epoxy | 0 | (4.5 ± 2.9) × 10$^{-12}$ | 1631.4 ± 91.8 | 138.1 ± 0.1 |
| GNP 2.5% T0 | 0 | (2.1 ± 0.5) × 10$^{-10}$ | 2648.7 ± 150.9 | 124.6 ± 1.8 |
| GNP 2.5% T120 UB | 3828 | (5.6 ± 4.5) × 10$^{-7}$ | 2761.8 ± 69.4 | 134.1 ± 0.9 |
| GNP 2.5% T15 A25 | 5700 | (6.8 ± 1.5) × 10$^{-10}$ | 2558.8 ± 50.0 | 126.4 ± 0.91 |
| GNP 2.5% T30 A25 | 12420 | (5.8 ± 1.3) × 10$^{-11}$ | 2603.1 ± 320.6 | 135.6 ± 0.13 |
| GNP 2.5% T45 A25 | 18500 | (5.9 ± 4.4) × 10$^{-10}$ | 2731.8 ± 278.5 | 130.0 ± 0.3 |
| GNP 2.5% T60 A25 | 25025 | (2.9 ± 2.2) × 10$^{-10}$ | 2709.8 ± 90.3 | 139.0 ± 1.3 |
| GNP 2.5% T15 A50 | 21450 | (7.0 ± 2.3) × 10$^{-11}$ | 2782.6 ± 95.8 | 128.2 ± 0.4 |
| GNP 2.5% T30 A50 | 43460 | (3.6 ± 0.9) × 10$^{-11}$ | 2751.1 ± 186.9 | 123.2 ± 2.1 |
| GNP 2.5% T45 A50 | 64530 | (3.8 ± 1.4) × 10$^{-11}$ | 2909.0 ± 48.2 | 126.5 ± 0.8 |
| GNP 2.5% T60 A50 | 87170 | (1.7 ± 0.7) × 10$^{-11}$ | 2849.2 ± 12.2 | 131.3 ± 0.4 |
| GNP 2.5% T15 A75 | 48100 | (7.6 ± 1.2) × 10$^{-11}$ | 2693.9 ± 301.2 | 105.8 ± 2.5 |
| GNP 2.5% T30 A75 | 90980 | (4.8 ± 0.7) × 10$^{-11}$ | 2324.2 ± 233.2 | 116.2 ± 1.6 |
| GNP 2.5% T45 A75 | 132700 | (2.4 ± 0.2) × 10$^{-11}$ | 2715.7 ± 106.5 | 133.7 ± 0.1 |
| GNP 2.5% T60 A75 | 171000 | (2.0 ± 0.3) × 10$^{-11}$ | 2654.3 ± 153.3 | 133.3 ± 2.2 |
| CNT 0.25% T0 | 0 | (1.1 ± 0.9) × 10$^{-4}$ | 2554.8 ± 133.1 | 132.1 ± 0 |

| | | | | |
|---|---|---|---|---|
| CNT 0.25% 120 UB | 3828 | (1.6 ± 1.3) × $10^{-5}$ | 2346.6 ± 81.7 | 133.4 ± 0.9 |
| CNT 0.25% T15 A25 | 5700 | (1.3 ± 0.1) × $10^{-3}$ | 2248.8 ± 5.9 | 128.8 ± 1.4 |
| CNT 0.25% T30 A25 | 12420 | (1.1 ± 0.1) × $10^{-3}$ | 2387.0 ± 15.7 | 131.7 ± 0.7 |
| CNT 0.25% T45 A25 | 18500 | (1.4 ± 0.1) × $10^{-3}$ | 2230.8 ± 52.3 | 114.7 ± 0.3 |
| CNT 0.25% T60 A25 | 25025 | (1.4 ± 0.2) × $10^{-3}$ | 2457.6 ± 40.7 | 128.3 ± 0.3 |
| CNT 0.25% T15 A50 | 21450 | (6.5 ± 0.4) × $10^{-4}$ | 2241.4 ± 74.1 | 132.0 ± 0.8 |
| CNT 0.25% T30 A50 | 43460 | (2.3 ± 0.4) × $10^{-4}$ | 2167.1 ± 91.0 | 110.2 ± 1.2 |
| CNT 0.25% T45 A50 | 64530 | (1.5 ± 0.1) × $10^{-4}$ | 1770.7 ± 25.98 | 100.1 ± 0.9 |
| CNT 0.25% T60 A50 | 87170 | (1.2 ± 0.2) × $10^{-4}$ | 1878.2 ± 85.7 | 82.9 ± 0.5 |
| CNT 0.25% T15 A75 | 48100 | (1.6 ± 0.6) × $10^{-3}$ | 2386.6 ± 82.0 | 122.8 ± 0.5 |
| CNT 0.25% T30 A75 | 90980 | (5.2 ± 1.4) × $10^{-4}$ | 2348.2 ± 22.4 | 121.3 ± 0.8 |
| CNT 0.25% T45 A75 | 132700 | (3.8 ± 0.6) × $10^{-4}$ | 2292.2 ± 5.7 | 116.7 ± 1.8 |
| CNT 0.25% T60 A75 | 171000 | (3.3 ± 0.4) × $10^{-4}$ | 2287.3 ± 112.6 | 112.5 ± 0.3 |
| HB 2.5/0.25% T0 | 0 | (4.2 ± 3.6) × $10^{-5}$ | 2931.4 ± 170.2 | 133.5 ± 1.0 |
| HB 2.5/0.25% T120 UB | 3828 | (6.7 ± 3.1) × $10^{-4}$ | 2660.2 ± 353.4 | 136.1 ± 2.8 |
| HB 2.5/0.25% T15 A25 | 5700 | (1.3 ± 1.2) × $10^{-3}$ | 2672.3 ± 165.8 | 137.5 ± 1.3 |
| HB 2.5/0.25% T30 A25 | 12420 | (5.9 ± 2.3) × $10^{-5}$ | 2719.3 ± 487.7 | 134.8 ± 0.4 |
| HB 2.5/0.25% T45 A25 | 18500 | (1.3 ± 0.8) × $10^{-3}$ | 2659.1 ± 13.7 | 135.9 ± 1.2 |
| HB 2.5/0.25% T60 A25 | 25025 | (4.6 ± 2.5) × $10^{-4}$ | 2994.5 ± 3.6 | 137.9 ± 0.5 |
| HB 2.5/0.25% T15 A50 | 21450 | (5.7 ± 0.2) × $10^{-6}$ | 2589.7 ± 223.8 | 133.4 ± 0.7 |
| HB 2.5/0.25% T30 A50 | 43460 | (3.2 ± 0.2) × $10^{-5}$ | 2749.9 ± 23.9 | 135.1 ± 0.3 |
| HB 2.5/0.25% T45 A50 | 64530 | (9.9 ± 7.0) × $10^{-6}$ | 2839 ± 180.7 | 138.4 ± 0.0 |
| HB 2.5/0.25% T60 A50 | 87170 | (6.0 ± 0.6) × $10^{-6}$ | 2817.5 ± 1.8 | 140.3 ± 1.1 |
| HB 2.5/0.25% T15 A75 | 48100 | (1.3 ± 0.3) × $10^{-5}$ | 2850.7 ± 60.6 | 132.6 ± 0.9 |
| HB 2.5/0.25% T30 A75 | 90980 | (1.2 ± 0.9) × $10^{-5}$ | 2492.5 ± 246.5 | 119.7 ± 1.0 |
| HB 2.5/0.25% T45 A75 | 132700 | (7.3 ± 2.7) × $10^{-6}$ | 2707.1 ± 79.7 | 134.3 ± 1.4 |
| HB 2.5/0.25% T60 A75 | 171000 | (8.9 ± 4.2) × $10^{-6}$ | 2824.0 ± 12.0 | 136.4 ± 0.5 |
